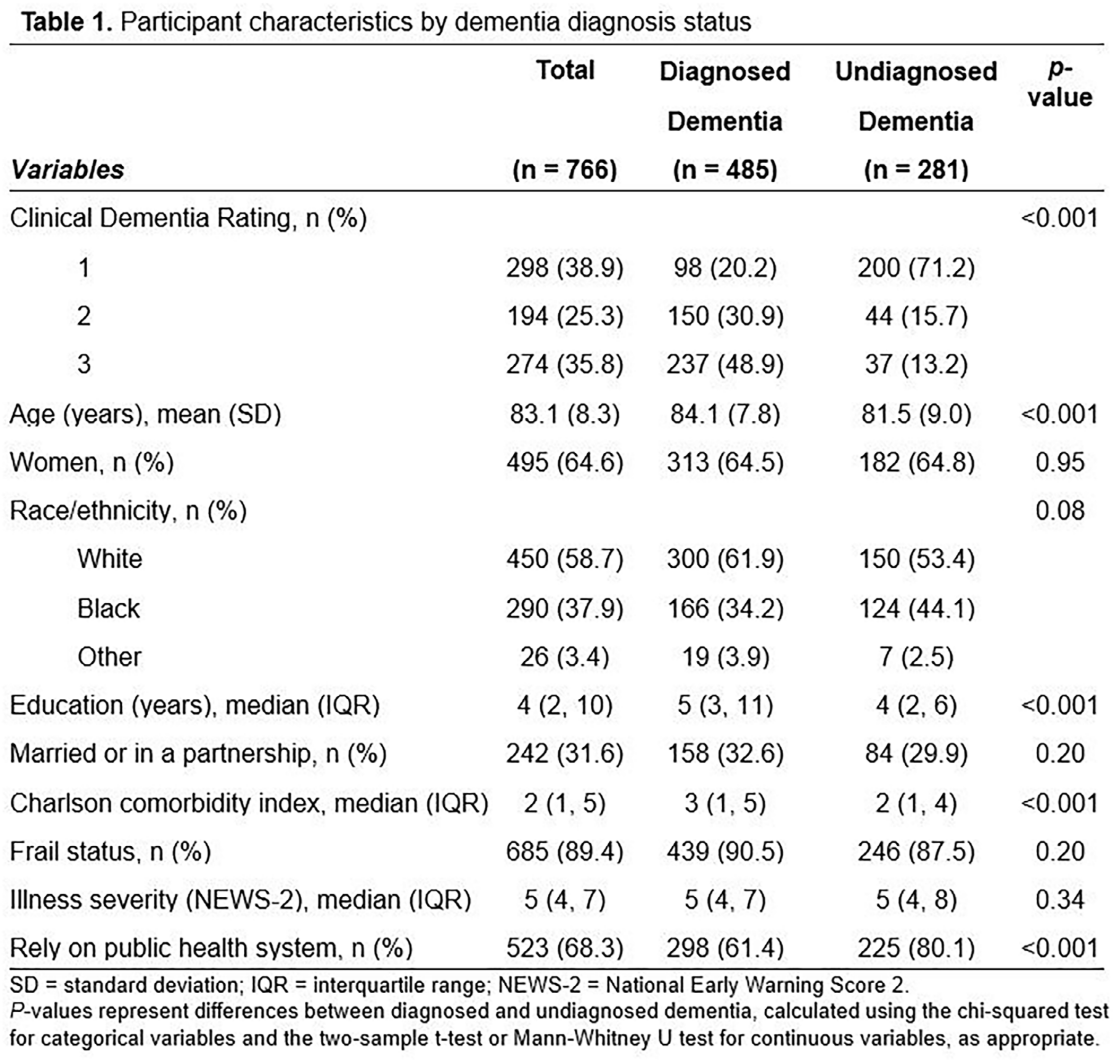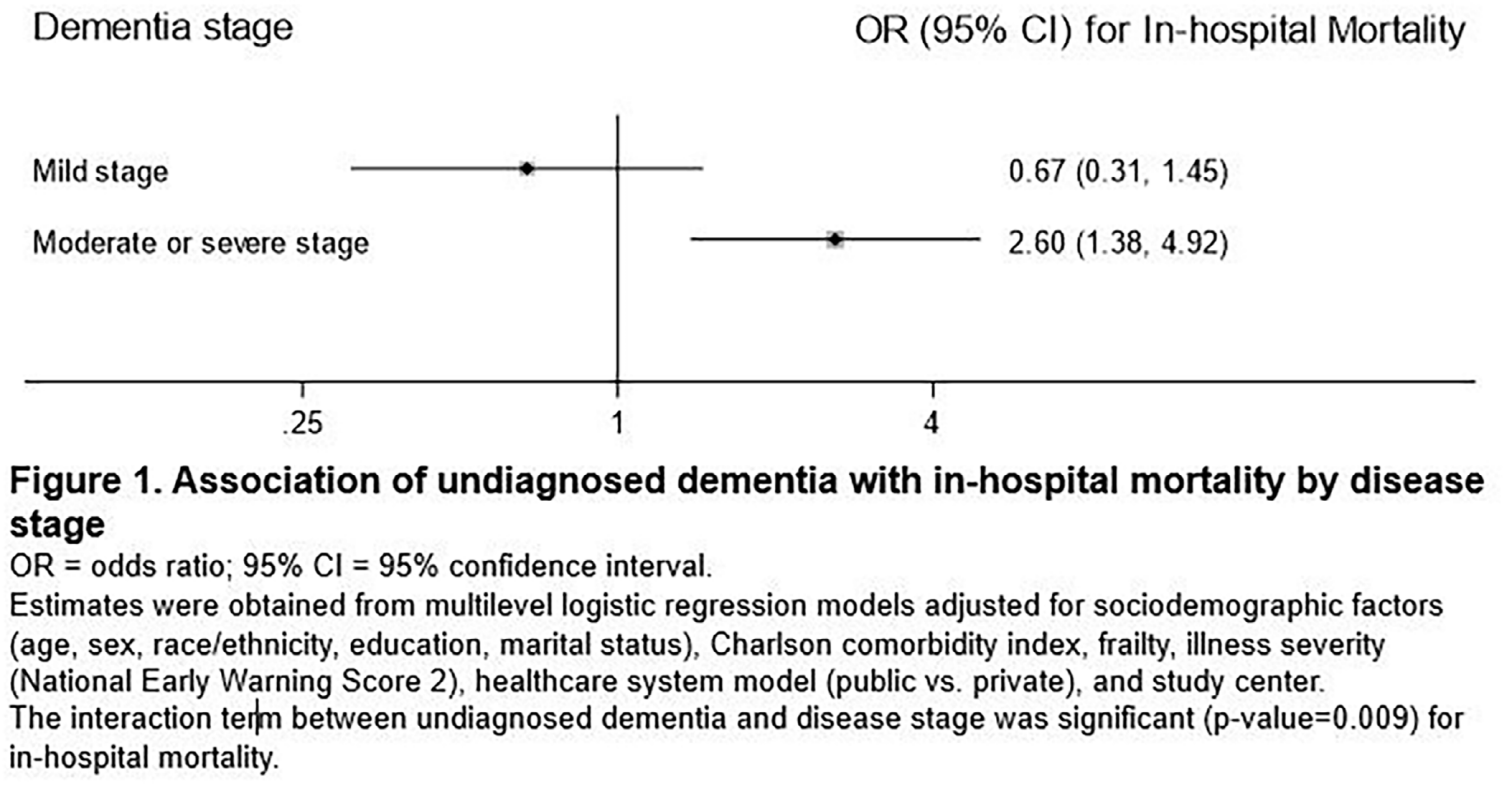# Before it is too late: undiagnosed dementia and mortality risks by disease stage in diverse acute care settings

**DOI:** 10.1002/alz70860_107512

**Published:** 2025-12-23

**Authors:** Márlon Juliano Romero Aliberti, Thiago J Avelino‐Silva, Kenneth E Covinsky, Claudia Kimie Suemoto

**Affiliations:** ^1^ University of São Paulo Medical School, São Paulo, São Paulo, Brazil; ^2^ Hospital Sírio‐Libanês, São Paulo, São Paulo, Brazil; ^3^ Johns Hopkins Bloomberg School of Public Health, Baltimore, MD, USA; ^4^ University of California San Francisco, San Francisco, CA, USA

## Abstract

**Background:**

Although dementia impacts acute care, the consequences of underdiagnosis across all disease stages remain unclear. Using data from an admixed population of older adults with dementia admitted to hospitals in five countries, we evaluated whether the association between undiagnosed dementia and in‐hospital mortality varies by disease stage.

**Methods:**

We conducted a prospective cohort of patients aged ≥65 years with dementia admitted to 43 acute hospitals in Brazil, Angola, Chile, Colombia, and Portugal. Upon admission, patients underwent a comprehensive geriatric assessment. Trained investigators administered the Clinical Dementia Rating (CDR) to close family informants, referencing cognitive status three months before admission (CDR=1 for mild, CDR=2 for moderate, and CDR=3 for severe dementia) to avoid the influence of acute cognitive impairments. To determine a previous dementia diagnosis, we reviewed medical records and consulted family. Undiagnosed dementia was defined as no previous dementia diagnosis and a CDR ≥1. We used multilevel logistic regression models to assess the association between undiagnosed dementia and in‐hospital mortality, incorporating interaction terms for dementia stage and adjusting for sociodemographic factors, comorbidities, frailty, illness severity, healthcare system model, and study center.

**Results:**

Among 766 patients with dementia (mean age=83±8 years; women = 65%; White=59%), 281 (37%) had no prior diagnosis. Undiagnosed dementia was more frequent in mild stage (67%) than in moderate or severe stages (17%). Compared to those with a prior diagnosis, patients with undiagnosed dementia were more likely to have lower education levels, fewer chronic disease diagnoses, and rely on public healthcare (Table 1). Overall, 140 (18%) patients died in the hospital. Undiagnosed dementia was not associated with increased mortality (20% vs. 17%; adjusted OR=1.47; 95%CI=0.90‐2.42). However, dementia stage modified this association (*p*‐value for interaction = 0.009). In mild stages, undiagnosed dementia was not linked to higher in‐hospital mortality. By contrast, in moderate or severe stages, undiagnosed dementia nearly tripled the odds of mortality compared to those with a prior diagnosis (Figure 1).

**Conclusion:**

While most undiagnosed dementia cases occur in mild stages, their impact on outcomes worsens with disease progression. Addressing socioeconomic disparities and strengthening healthcare systems are essential to improving diagnosis, acute care, and outcomes for patients with dementia.